# Induction of Chronic Inflammation and Altered Levels of DNA Hydroxymethylation in Somatic and Germinal Tissues of CBA/CaJ Mice Exposed to ^48^Ti Ions

**DOI:** 10.3389/fonc.2016.00155

**Published:** 2016-06-27

**Authors:** Kanokporn Noy Rithidech, Witawat Jangiam, Montree Tungjai, Chris Gordon, Louise Honikel, Elbert B. Whorton

**Affiliations:** ^1^Department of Pathology, Stony Brook University, Stony Brook, NY, USA; ^2^Department of Chemical Engineering, Faculty of Engineering, Burapha University, Chonburi, Thailand; ^3^Department of Radiologic Technology, Faculty of Associated Medical Sciences, Center of Excellence for Molecular Imaging, Chiang Mai University, Chiang Mai, Thailand; ^4^StatCom, Galveston, TX, USA

**Keywords:** titanium ions, chronic inflammation, NF-ĸB, pro-inflammatory cytokines, lung, testes, 5-methylcytosine, 5-hydroxymethylcytosine

## Abstract

Although the lung is one of the target organs at risk for cancer induction from exposure to heavy ions found in space, information is insufficient on cellular/molecular responses linked to increased cancer risk. Knowledge of such events may aid in the development of new preventive measures. Furthermore, although it is known that germinal cells are sensitive to X- or γ-rays, there is little information on the effects of heavy ions on germinal cells. Our goal was to investigate *in vivo* effects of 1 GeV/n ^48^Ti ions (one of the important heavy ions found in the space environment) on somatic (lung) and germinal (testis) tissues collected at various times after a whole body irradiation of CBA/CaJ mice (0, 0.1, 0.25, or 0.5 Gy, delivered at 1 cGy/min). We hypothesized that ^48^Ti-ion-exposure induced damage in both tissues. Lung tissue was collected from each mouse from each treatment group at 1 week, 1 month, and 6 months postirradiation. For the testis, we collected samples at 6 months postirradiation. Hence, only late-occurring effects of ^48^Ti ions in the testis were studied. There were five mice per treatment group at each harvest time. We investigated inflammatory responses after exposure to ^48^Ti ions by measuring the levels of activated nuclear factor kappa B and selected pro-inflammatory cytokines in both tissues of the same mouse. These measurements were coupled with the quantitation of the levels of global 5-methylcytosine (5mC) and 5-hydroxymethylcytosine (5hmC). Our data clearly showed the induction of chronic inflammation in both tissues of exposed mice. A dose-dependent reduction in global 5hmC was found in the lung at all time-points and in testes collected at 6 months postirradiation. In contrast, significant increases in global 5mC were found only in lung and testes collected at 6 months postirradiation from mice exposed to 0.5 Gy of 1 GeV/n ^48^Ti ions. Overall, our data showed that ^48^Ti ions may create health risks in both lung and testicular tissues.

## Introduction

Spaceflight results in unavoidable exposure of astronauts to space radiation (such as heavy ions and energetic protons) that may create potential risks for late-occurring injuries in both somatic and germinal cells/tissues. To protect astronauts, we must improve our understanding of changes at the cellular and molecular levels that are linked to increasing astronaut health risks and are valuable in developing countermeasures. In order to obtain reliable information about radiation-induced detrimental health effects, the data must be obtained using appropriate *in vivo* systems because *in vitro* systems cannot faithfully mimic the complex *in vivo* situation (1). Hence, appropriate whole-animal systems are critically important surrogates for assessment of health risks associated with exposure to space radiation.

The aim of this study was to improve our knowledge of *in vivo* biological effects of a whole body exposure to 1 GeV/n ^48^Ti ions (one of the important types of heavy ions found in the space environment). We used the CBA/CaJ mouse as an experimental model to study the effects of 1 GeV/n ^48^Ti ions on the lung (representing somatic tissue) and the testis (representing germinal tissue) of the same mouse, employing the inflammatory responses and DNA methylation endpoints. These two endpoints have not been used to evaluate the biological effects of 1 GeV/n ^48^Ti ions in somatic and germinal cells of the same mouse, setting our approach apart from the existing reports.

It is known that the lung is a highly radiosensitive organ (2–4) and that impairment of the immune function in the lung is one of the major concerns after exposure to low LET radiation (4–7). It also has been suggested that the lung is one of the target organs at risk for cancer induction from exposure to heavy ions found in space (8). However, very little is known about the responses of the lung to space radiation. Recently, it was found that 350 MeV/n ^28^Silicon (^28^Si) or ^56^Iron (^56^Fe) ions, which are also important heavy ions found in the space environment, induced both histological and functional injuries in the lungs of exposed C3H/HeNCrl mice (9). Furthermore, it was reported that 1 GeV/n ^56^Fe ions induced lung cancer in transgenic mice (the Kras^LA1^ mice) engineered to be susceptible to lung cancer (10, 11).

With respect to testes, deleterious effects (e.g., DNA double-strand breaks, cytogenetic effects, and mutagenesis) of X or γ rays on spermatogenesis were reported several decades ago (12–19). It is known that the testis is one of the most radiosensitive organs (20) and is more sensitive to radiation exposure than female germ cells (21). Thus, male-mediated reproductive and developmental toxicology has been a concern for decades in atomic bomb survivors and in the Sellafield nuclear plant workers (22). However, very little is known about the effects of heavy ions on testes. It was found that exposure to 2.0–8.07 Gy of 0.35 GeV/n ^12^Carbon (^12^C) ions (LET = 13 keV/μm) or to 0.3–2.0 Gy of 1 GeV/n ^56^Fe ions (LET = 147 keV/μm) did not increase mutation rates (assayed by the specific locus and the dominant lethal tests in Medaka fish), as compared to those exposed to 250 kVp X rays, a reference radiation (22). However, prenatal irradiation of pregnant rats to 0.1–2.0 Gy of 0.3 GeV/n ^12^C or to 0.1–0.5 Gy of 0.4 MeV/n ^20^Neon (^20^Ne) ions (LET = 30 keV/μm) caused abnormal testicular development and breeding activity of male offspring (23). Further, an increased level of interleukin-1β, lower number of sperms, and an abnormal tubular architecture were found in testes of C57BL/6 mice flown with the Space Shuttle Discovery for 114 days, in relation to that of the corresponding sham controls (with no spaceflight) (24). Of note, it is known that the space environment is complex. Several factors (e.g., radiation, microgravity, and reactivation of herpes virus infection) may have contributed to such changes. Hence, to reduce the uncertainties in the assessment of health risks of space radiation, further ground-based studies are required to help improve our understanding of the effects of heavy ions on germinal cells and somatic cells as well.

Currently, there is no information on the *in vivo* effects of 1 GeV/n ^48^Ti ions to the lung and the testes. Based upon the existing, but limited, information on responses to 1 GeV/n ^48^Ti ions (25–27), we hypothesized that 1 GeV/n ^48^Ti ions induced damage to these two tissues of exposed mice. To address this hypothesis, we used two biological endpoints to evaluate the effects of 1 GeV/n ^48^Ti ions on lung and testicular tissues of the same mouse. These endpoints were inflammatory responses and global DNA methylation, including both 5-methylcytosine (5mC) and 5-hydroxymethylcytosine (5hmC). These two biological endpoints were chosen for analyses because they are highly relevant surrogate biomarkers for assessing health risks, but they have not previously been assessed following *in vivo* exposure to 1 GeV/n ^48^Ti ions. Importantly, the induction of chronic inflammation has been reported in studies of astronauts’ blood samples (28–30).

There has been increasing evidence of space-radiation-induced acute and chronic inflammation (26, 31–35), and radiation-induced aberrant DNA methylation at the global (26, 36) or specific locus levels (37–41). For the inflammatory responses, in this report, we chose to study the nuclear-factor kappa B (NF-κB) pathway because NF-κB is a key transcription factor playing a pivotal role in inflammatory responses to oxidative stress induced by several stimuli, including radiation (42). Although NF-κB is a member of the ubiquitously expressed family of the Rel-related transcription factors (43), only the activation of NF-κB/p65 was the focus of our study and referred to as NF-κB throughout the article. It also has been well recognized that NF-κB is a key transcription factor known to be part of a common network between inflammation and cancer (44–46), and that there is a close association between inflammation and cancer (44, 47–54). In addition, chronic inflammation in male germinal cells has been linked to male infertility (55–57). In addition to the levels of activated NF-κB, we measured the expression, at the protein levels, of selected NF-κB-regulated pro-inflammatory cytokines, i.e., tumor necrosis factor alpha (TNF-α), interleukin-1 beta (IL-1β), and interleukin 6 (IL-6). This is because their increased levels have been found in the liver (26) of the same exposed mice included in this present study. Furthermore, the expression of these proteins (at the gene level) was elevated in human mononuclear cells obtained from healthy adult individuals who lived near the Chernobyl Nuclear Power Plant and were chronically exposed to low-dose radiation ranging from 0.18 to 49 mSv (58).

Relating to DNA methylation, it has been well recognized that it is one of the key epigenetic events that plays a critical role in carcinogenesis, both initiation and promotion, in somatic and germinal cells (59, 60), and other untoward health outcomes (14) including male-mediated developmental toxicology (61), male infertility (62–64), and transgeneration effects (65, 66). Furthermore, a high level of 5mC (hypermethylation) has been linked to gene silencing (59, 67); while a reduction in global levels of 5hmC has been associated with cancer development (68). Since inflammatory responses and DNA methylation were analyzed in the lung and the testes of the same mouse, it is possible to investigate the differential sensitivity of these two tissues in the same mouse.

## Materials and Methods

### Animals

The CBA/CaJ mice included in this study were the same cohort that were used to investigate the effects of 1 GeV/n ^48^Ti ions on the liver previously reported (26), where the description of the CBA/CaJ mice, ^48^Ti-irradiation, and animal husbandry were presented. The experimental design of the study was approved by both the Brookhaven National Laboratory (BNL) and the Stony Brook University (SBU) Institutional Animal Care and Use Committee (IACUC). Of note, the CBA/CaJ mouse is known to be sensitive to the development of radiation-induced myeloid leukemia (ML) (69–76), liver cancer (hepatocellular carcinoma or HCC) (70, 75), and lung cancer (70).

### Irradiation of Mice

**Figure 1:**
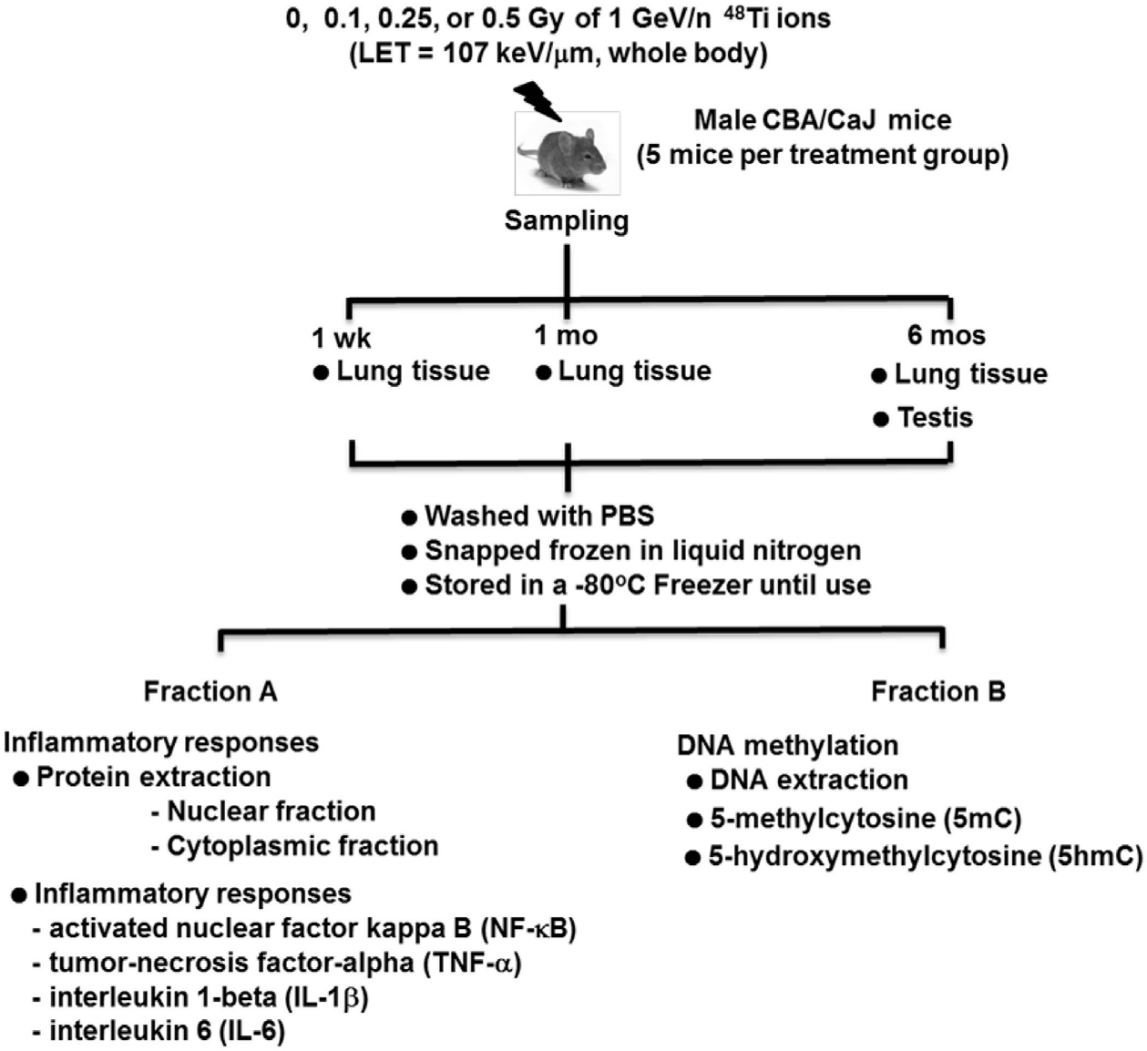
**Diagram of the experimental design**.

Figure 1 is a diagram of the experimental design. Mice were exposed, whole-body, to average total-body doses of 0, 0.1, 0.25, or 0.5 Gy of 1 GeV/n ^48^Ti ions, delivered at a dose rate of 0.01 Gy/min by a 20 cm × 20 cm beam. Mice included in the sham-control group (i.e., those that were exposed to 0 Gy) were age-matched to exposed mice. Therefore, the age of mice in each treatment group would be similar at each sacrifice time. The exposure of mice was done at the National Aeronautics and Space Administration (NASA) Research Laboratory (NSRL) located at BNL. Details of the NSRL facility and irradiation procedure were previously provided (26, 34, 35, 77). We designated the first day after irradiation as day 1 after exposure. Mice were transported to the animal facility of SBU in a climate-controlled vehicle within 2 days postirradiation. Similar to the animal facility located in BNL, the animal facility of SBU, where sample collections were performed, is also approved by the Association for Assessment and Accreditation of Laboratory Animal Care (AAALAC), with the same light cycle (12 h light/12 h dark), temperature (21 ± 2°C), 10–15 hourly cycles of fresh air, and relative humidity (50 ± 10%).

### Collection of the Lung and Testis

For the lung, groups of mice were used for sampling at 1 week, 1 month, and 6 months following the exposure to 1 GeV/n ^48^Ti ions. In contrast, the collection of the testis was done at 6 months postexposure only. Hence, at 6 months postirradiation, the lung and the testis were collected from the same mouse. The rational for choosing only one harvest time for the testis was because our goal was to study the effects of ^48^Ti ions on the stem-cell compartment of spermatogenesis. It is known that spermatogenesis is a complex biological process involving the transformation of spermatogonial stem cells (types A and B) into primary and secondary spermatocytes, round spermatids, and, eventually, spermatozoa over an extended period of time within seminiferous tubules of the testis (60, 65, 78). The duration of mouse spermatogenesis from the primitive type A_single_ spermatogonial stem cells (SSCs) to mature sperms (spermatozoa) is about 52 days, but 35 days from differentiated spermatogonia to mature sperms (62, 79). Hence, the results obtained from the analyses of the testes collected at 6 months postirradiation reflect the effects of radiation on type A_single_ SSCs.

From each treatment group, we collected the tissues from each mouse (five mice per dose of ^48^Ti ions). Briefly, the lung and testicular tissues were removed, rinsed three times with 1 mL phosphate buffered saline (PBS) each time to remove external contamination (i.e., blood), snap frozen in liquid nitrogen, and stored in a −80°C freezer until needed for protein extraction and further analyses. After thawing, the total lung tissue was homogenized using a Bullet Blender Homogenizer (Next Advance Inc., Averill Park, NY, USA). Likewise, after thawing, the epididymis was removed from each testis to obtain seminiferous tubules, which were homogenized for use in protein extraction. The protocols for protein extraction from the lung and the testis suggested by the manufacturer were followed. Then, the cell lysates from each tissue of each mouse were divided into two fractions, i.e., fractions A and B. Fraction A of the tissue lysate was used to extract proteins from nuclear and cytosolic samples using the method we previously described (26, 34, 35, 80, 81). The total protein obtained from the nuclear portion of the lysate suspension of the lung or the testis was used for measuring the levels of NF-κB, while the total protein obtained from the cytosolic portion was used for the measurements of NF-κB-regulated pro-inflammatory cytokines, i.e., TNF-α, IL-1β, and IL-6. Protein contents in the cytosolic portion and the nuclear portion of the lung or the testis were measured by the Bradford assay using a BioPhotometer (Eppendorf, Inc., Westbury, NY, USA). Fraction B of the tissue lysate was used to isolate DNA for the measurements of global 5mC and global 5hmC.

### Measurement of Activated Nuclear Factor-Kappa B

As with our previous work (26, 34, 35, 81), we used the enzyme-linked immunosorbent assay (ELISA) NF-κB kits from Active Motif North America, Inc. for measuring the levels of activated NF-κB in the nuclear portions obtained from the lung and testis lysates. The assay was performed in duplicate wells for each lung or testis sample of each treatment group. The mean value of activated NF-κB levels for the tissue of each mouse was obtained and reported. Then, the mean value of ten measurements from five mice and the SE for each treatment group were obtained.

### Measurement of NF-κB-Regulated Pro-Inflammatory Cytokines, Including Tumor Necrosis Factor-α, Interleukin-1β, and Interleukin-6

Coupled with the levels of activated NF-κB, we measured the expression (at the protein levels) of NF-κB-regulated pro-inflammatory cytokines, i.e., TNF-α, IL-1β, and IL-6. The rational for studying these pro-inflammatory cytokines has been presented in the Section “Introduction.” We applied the methods routinely used in our laboratory for measuring the expression levels of these selected cytokines in lung or testicular cell suspensions (the cytosolic portions) using the specific ELISA kits for TNF-α, IL-1β, and IL-6 from Biosource (Invitrogen, Carlsbad, CA, USA) (26, 34, 35, 82). The mean value and SE of each cytokine for each treatment group were calculated from the means of five mice.

### Measurement of Global 5-Methylcytosine and 5-Hydroxymethylcytosine

The methods for DNA isolation from mouse tissues have previously been presented (26, 36). Commercially available ELISA kits for the detection of global 5mC and 5hmC (Zymo Research, Inc., Irvine, CA, USA) were used to measure the percentage of global 5mC and 5hmC in the DNA samples isolated from lung or testicular tissues. The levels of global 5mC and 5hmC were measured using a microplate spectrophotometer (Molecular Devices) at 405 nm. The % 5mC and % 5hmC was calculated from a standard curve generated using the control DNA set provided by the manufacturer. The measurements of 5mC and 5hmC in the DNA sample from each tissue of each mouse were done in duplicate (using 200 ng of DNA per well). Then, the mean value of global 5mC and global 5hmC for each mouse were obtained. Finally, the mean value of ten measurements and SE of global 5mC and global 5hmC for each treatment group were calculated from the means of five mice.

### Statistical Analyses

We expressed levels of each biological endpoint as mean ± SE. For each tissue, the mean value for each assay of each mouse was used as a single datum point for statistical analyses. At each harvest time, an ANOVA method appropriate for a one-factor experiment (i.e., dose of 1 GeV/n ^48^Ti ions) was used to assess the significance of the radiation dose. Further, the Student’s *t*-test was used, independently, to evaluate statistical differences in the mean values between each exposed group and the corresponding sham-control group. A *P*-value of ≤0.05 was considered as statistically significant.

## Results

**Figure 2:**
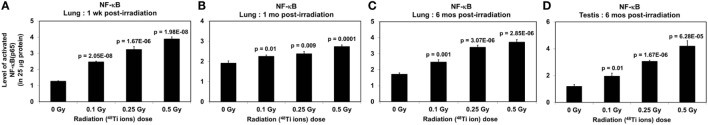
**Levels of activated NF-ĸB (±SE) in lung tissues collected at 1 week (A), 1 month (B), 6 months (C), and in testicular tissues collected at 6 months (D) from CBA/CaJ mice after a whole body exposure to various doses of 1 GeV/n ^48^Ti ions**. *P* values (Student’s *t*-test) indicate significant differences in the levels of NF-κB between exposed and corresponding sham control groups.

**Figure 3:**
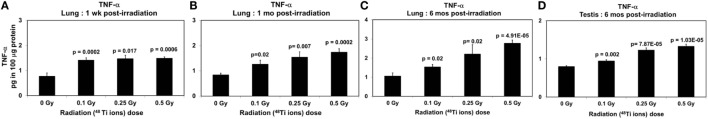
**Levels of TNF-α (±SE) in lung tissues collected at 1 week (A), 1 month (B), 6 months (C), and in testicular tissues collected at 6 months (D) from CBA/CaJ mice after a whole body exposure to various doses of 1 GeV/n ^48^Ti ions**. *P* values (Student’s *t*-test) indicate significant differences in the levels of TNF-α between exposed and corresponding sham control groups.

**Figure 4:**
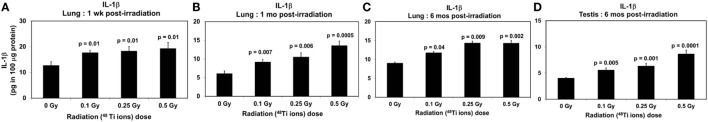
**Levels of IL-1β (±SE) in lung tissues collected at 1 week (A), 1 month (B), 6 months (C), and in testicular tissues collected at 6 months (D) from CBA/CaJ mice after a whole body exposure to various doses of 1 GeV/n ^48^Ti ions**. *P* values (Student’s *t*-test) indicate significant differences in the levels of IL-1β between exposed and corresponding sham control groups.

**Figure 5:**
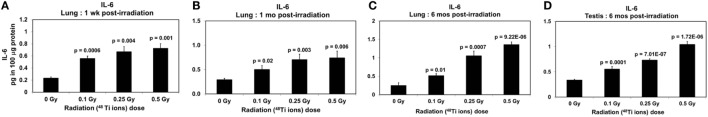
**Levels of IL-6 (±SE) in lung tissues collected at 1 week (A), 1 month (B), 6 months (C), and in testicular tissues collected at 6 months (D) from CBA/CaJ mice after a whole body exposure to various doses of 1 GeV/n ^48^Ti ions**. *P* values (Student’s *t*-test) indicate significant differences in the levels of IL-6 between exposed and corresponding sham control groups.

**Figure 6:**
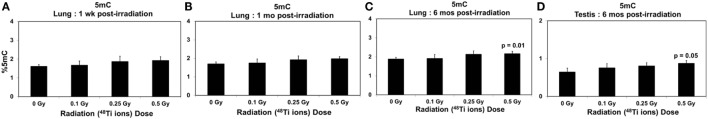
**Levels of 5mC (±SE) in lung tissues collected at 1 week (A), 1 month (B), 6 months (C), and in testicular tissues collected at 6 months (D) from CBA/CaJ mice after a whole body exposure to various doses of 1 GeV/n ^48^Ti ions**. *P* values (Student’s *t*-test) indicate significant differences in the levels of 5mC between exposed and corresponding sham control groups.

**Figure 7:**
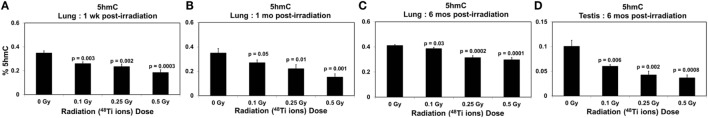
**Levels of 5hmC (±SE) in lung tissues collected at 1 week (A), 1 month (B), 6 months (C), and in testicular tissues collected at 6 months (D) from CBA/CaJ mice after a whole body exposure to various doses of 1 GeV/n ^48^Ti ions**. *P* values (Student’s *t*-test) indicate significant differences in the levels of 5hmC between exposed and corresponding sham control groups.

Figures 2–7 show the effects of various doses of 1 GeV/n ^48^Ti ions on the lung and testicular tissues of exposed CBA/CaJ mice. *P* values (Student’s *t*-test) shown in each figure indicate statistically significant levels between exposed and sham-control groups. Tables 1 and 2 show the results of the ANOVA for the lung and testicular tissues, respectively.

**Table 1:** **Analysis of variance results for lung tissues collected at 1 week, 1 month, and 6 months postirradiation, respectively (SS, sum of squares; df, degree of freedom; MS, mean of squares; *F, F*-statistic)**.

1 Week postirradiation						1 Month week postirradiation						6 Months postirradiation					
*Source of variation*	SS	df	MS	F	*P*-value	*Source of variation*	SS	df	MS	F	*P*-value	*Source of variation*	SS	df	MS	F	*P*-value
**NF-ĸB**																	
Between groups	18.95	3	6.32	102.1	1E-10	Between groups	1.74	3	0.58	12.6	0.0001	Between groups	12.44	3	4.15	43.86	6E-08
Within groups	0.99	16	0.06			Within groups	0.74	16	0.05			Within groups	1.51	16	0.09		
Total	19.94	19				Total	2.48	19				Total	13.95	19			
**TNF-α**																	
Between groups	1.78	3	0.59	5.25	0.01	Between groups	2.27	3	0.76	6.31	0.0049	Between groups	8.55	3	2.85	7.15	0.002
Within groups	1.81	16	0.11			Within groups	1.92	16	0.12			Within groups	6.37	16	0.40		
Total	3.59	19				Total	4.19	19				Total	14.92	19			
**IL-1β**																	
Between groups	128.91	3	42.97	2.86	0.04	Between groups	144.64	3	48.21	9.67	0.0007	Between groups	62.52	3	20.84	6.86	0.003
Within groups	240.68	16	15.04			Within groups	79.74	16	4.98			Within groups	48.58	16	3.04		
Total	369.59	19				Total	224.38	19				Total	111.09	19			
**IL-6**																	
Between groups	0.51	3	0.17	7.62	0.0029	Between groups	0.64	3	0.21	4.37	0.02	Between groups	3.50	3	1.17	29.49	2E-06
Within groups	0.31	16	0.02			Within groups	0.78	16	0.05			Within groups	0.59	16	0.04		
Total	0.82	19				Total	1.43	19				Total	111.09	19			
**5mC**																	
Between groups	0.34	3	0.11	0.49	0.68	Between groups	0.26	3	0.09	0.39	0.76	Between groups	0.61	3	0.20	1.65	0.22
Within groups	3.64	16	0.23			Within groups	3.61	16	0.23			Within groups	1.99	16	0.12		
Total	3.98	19				Total	3.87	19				Total	2.61	19			
**5hmC**																	
Between groups	0.07	3	0.02	11.38	0.0003	Between groups	0.05	3	0.02	7.29	0.003	Between groups	0.05	3	0.02	16.15	4E-05
Within groups	0.03	16	0.002			Within groups	0.01	16	0.0050			Within groups	0.01	16	0.0009		
Total	0.1	19				Total	0.06	19				Total	0.06	19			

**Table 2:** **Analysis of variance results for testicular tissues collected at 6 months postirradiation (SS, sum of squares; df, degree of freedom; MS, mean of squares; *F, F*-statistic)**.

Source of variation	SS	df	MS	*F*	*P*-value
**NF-kB**					
Between groups	25.70	3	8.57	27.39	1.5379E-06
Within groups	5.00	16	0.31		
Total	30.70	19			
**TNF-α**					
Between groups	0.91	3	0.30	29.46	9.4514E-07
Within groups	0.16	16	0.01		
Total	1.07	19			
**IL1-β**					
Between groups	56.46	3	18.82	14.45	8.1361E-05
Within groups	20.85	16	1.30		
Total	77.31	19			
**IL-6**					
Between groups	1.34	3	0.45	49.31	2.6281E-08
Within groups	0.14	16	0.01		
Total	1.48	19			
**5mC**					
Between groups	0.16	3	0.05	1.19	0.35
Within groups	0.72	16	0.05		
Total	0.88	19			
**5hmC**					
Between groups	0.01	3	0.0042	12.88	0.0001
Within groups	0.01	16	0.0003		
Total	0.02	19			

### Activated Nuclear Factor-Kappa B

There were dose-dependent increases in the levels of activated NF-κB in lung tissues from all exposed groups (ANOVA, *P* < 0.01), regardless of the harvest time (Figures 2A–C). Of note, there is a fluctuation in the levels of activated NF-κB in lung tissues collected from sham control mice (in particular in those collected at 1 month postirradiation). However, the factors contributing to this temporal change are unknown. We also detected a dose-dependent increase in the levels of activated NF-κB in the testicular tissues (ANOVA, *P* < 0.01) at 6 months postirradiation (as shown in Figure 2D).

### Tumor Necrosis Factor-α

Similar to the levels of activated NF-κB, there were dose-dependent increases in the level of TNF-α in lung tissues collected at all time-points (ANOVA, *P <* 0.01), as shown in Figures 3A–C. Likewise, at 6 months postirradiation, a dose-dependent increase (ANOVA, *P* < 0.01) in the levels of IL-6 was found in testicular tissues collected at 6 months postirradiation (Figure 3D).

### Interleukin-1β and Interleukin-6

Clearly, dose-dependent increases in the levels of IL-1β (Figures 4A–C) and IL-6 (Figures 5A–C) were observed in lung tissues collected at 1 week, 1 month, and 6 months postirradiation (ANOVA, *P* < 0.01), respectively. Of note, similar to activated NF-κB, there was a fluctuation in the levels of IL-1β in lung tissues of sham control mice, in particular in those collected at 1 month postirradiation. The cause of such fluctuation remains unidentified. Likewise, dose-dependent increases in the levels of IL-1β (Figure 4D) and IL-6 (Figure 5D) in testicular tissues collected at 6 months postirradiation (ANOVA, *P* < 0.01) were evident.

### 5 -Methylcytosine and 5-Hydroxymethylcytosine

Figures 6A–C show the effects of ^48^Ti ions on the levels of global 5mC in lung tissues of exposed mice. There was a trend of increased levels of global 5mC in the lung tissues of exposed mice, in relation to those of the corresponding sham controls. However, such increases were not statistically different, except in the lung tissues collected at 6 months from mice exposed to the highest dose of 1 GeV/n ^48^Ti ions. A similar finding was found in testicular tissues collected at 6 months postirradiation (Figure 6D).

In contrast, Figures 7A–C show significant dose-dependent decreases in the levels of 5hmC in the lungs of exposed mice (ANOVA, *P* < 0.05) at 1 week, 1 month, and 6 months, respectively. The decreases in 5hmC levels in lung tissues of exposed mice relative to the corresponding sham controls were: 1.33-, 1.48-, and 1.88-fold at 1 week postirradiation; 1.29-, 1.58-, and 2.29-fold at 1 month postirradiation; 1.06-, 1.30-, and 1.38-fold at 6 months postirradiation. Likewise, there was a dose-dependent reduction in the levels of global 5hmC in testicular tissues collected at 6 months postirradiation as shown in Figure 7D.

## Discussion

Our data are the first to report the presence of chronic inflammation and altered levels of global 5hmC in the lung and testicular tissues of CBA/CaJ mice after a whole body exposure to 1 GeV/n ^48^Ti ions at low doses and a low dose-rate relevant to what is found in space, i.e., 0.1–0.5 Gy (delivered at 0.01 Gy/min). Our data also indicated that only 0.5 Gy (the highest dose used in our study) of 1 GeV/n ^48^Ti ions induced significant increases in the levels of global 5mC in both tissues of the same mouse. The magnitude of the effects of ^48^Ti ions on each tissue is similar. Since these two endpoints were detected in both tissues of the same mouse, it is plausible to speculate that there is a connection between chronic inflammation and altered DNA methylation. The information obtained from our study is important because these two *in vivo* endpoints are the hallmarks of cancer (52, 53, 68) and several types of male germ-cell disturbance (55–57, 62, 63, 83, 84). Hence, our findings provide an important foundation for future studies in which an association between molecular changes and the histopathological, pathological and/or functional damage in the lung and the testes, including the incidence of lung or testicular cancer, can be achieved. Of note, in future studies, it is important to measure the levels of activated NF-ĸB and related pro-inflammatory cytokines not only in tissues but also in plasma obtained from the same mice. The obtained information will help to determine whether there is a correlation between chronic inflammation in tissues and the levels of circulating cytokines, which should have clinical implications.

The approach we used in this study has allowed the investigation of the kinetics of effects of 1 GeV/n ^48^Ti ions on the lung, not only as a function of radiation dose but also time after exposure, since lung tissues were collected at various times up to 6 months postirradiation. We observed dose- and time-dependent increases in the levels of activated NF-κB and expression of NF-κB-related pro-inflammatory cytokines (i.e., TNF-α, IL-1β, and IL-6). Our data indicate that ^48^Ti-ion-exposure induces disturbances of cytokine production, reflecting chronic inflammation and an impairment of the immune system. Relating to the kinetics of the levels of global 5mC and 5hmC in the lung, our data indicated no significant change in the levels of global 5mC, except a significant increase in lung tissues collected at 6 months postirradiation from mice exposed to 0.5 Gy of ^48^Ti ions. In contrast, there were significant dose-dependent decreases in the levels of global 5hmC at all harvest time-points. Such findings were similar to those detected in the liver collected from the same mouse previously reported (26). Hence, our data suggest that the loss of global 5hmC is a significant response to ^48^Ti-ion-irradiation, regardless of tissue type. It also is reasonable to hypothesize that chronic inflammation enhances radiation-induced loss of global 5hmC and *vice versa*.

Our study is the first to report the levels of global 5hmC in the lung of mice exposed to radiation. Of note, the focus of previous studies on the effects of radiation, both low and high LET, has been on specific loci of 5mC (38, 39, 41, 85–88). We included the levels of global 5hmC because it is currently recognized that a reduction in global 5hmC is a biomarker for cancer (68). Taken together, our data suggest that a reduction in the level of global 5hmC may be a better hallmark of radiation exposure than an increased level of global 5mC. In the future, it will be important to conduct studies to determine the genome-wide profiling of 5hmC/5mC to reveal the affected regions of the genome so that, in turn, the identification of affected genes will be possible.

Regarding the testes collected at 6 months from the same mouse from which the lung tissue was collected for our study, the data clearly showed that there were dose-dependent increases in the levels of activated NF-κB and expression of NF-κB-regulated pro-inflammatory cytokines (i.e., TNF-α, IL-1β, and IL-6). These findings represent the effects of 1 GeV/n ^48^Ti ions on the primitive type of spermatogonial stem cells (SSCs), i.e., type A_single_ SSCs. The induced damage arising from exposed SSCs is highly relevant for genetic risk assessment since SSCs are capable of self-renewal and differentiation into spermatocytes and mature sperm. Hence, any induced damage in the SSC compartment, if not repaired, will be carried onto the next generation and will adversely impact self-renewal, proliferation, and differentiation. In contrast, the damage that is induced in other male germ cell stages (e.g., spermatocytes, where cell divisions, both in meiosis and mitosis, take place) will affect progenies that are conceived shortly after irradiation. Our new data are important because it has been well recognized that SSCs are responsible for long-term effects of radiation on fertility (15, 89). Further, it was suggested that inflammation in SSCs leads to failure of testicular androgen and sperm production, resulting in infertility (90), and testicular cancer (91). Thus, exposure to 1 GeV/n ^48^Ti ions may lead to health risks associated with the male reproductive system. At 6 months postirradiation, the effects of 1 GeV/n ^48^Ti ions on levels of global 5mC and 5hmC in the testes of exposed mice are similar to those found in the lung of the same mouse. As mentioned previously, altered DNA methylation plays an important role in male infertility (62–64), germ cell tumors (59, 60), and transgeneration effects (65, 78). Hence, our data provide critical information for conducting further studies to investigate the potential induction of these untoward outcomes on male germinal cells from exposure to 1 GeV/n ^48^Ti ions.

In summary, our results provide new information on *in vivo* biological responses to ^48^Ti ions. Our new data show that 1 GeV/n ^48^Ti ions (at doses ranging from 0.1 to 0.5 Gy, delivered at 0.01 Gy/min) can induce chronic inflammation, and a persistence of altered DNA methylation (at the global level) in lung and testicular tissues of exposed CBA/CaJ mice. Importantly, our findings provide an important foundation for further investigations on the genes/proteins involved in ^48^Ti-ion-induced chronic inflammation and altered DNA methylation. Knowing such detailed molecular markers for health risks from exposure to heavy ions would not only greatly improve radiation protection guidance for astronauts (or cancer patients receiving heavy-ion radiation therapy) but would also provide significantly valuable insight for developing biological countermeasures.

## Author Contributions

KR was responsible for the study concept and design; critical revision of the manuscript for important intellectual content. WJ was responsible for acquisition of the data. MT was responsible for acquisition of the data. CG was responsible for acquisition of the data. LH was responsible for acquisition of the data. EW was responsible for statistical analyses and critical revision of the manuscript for important intellectual content.

## Conflict of Interest Statement

The authors declare that the research was conducted in the absence of any commercial or financial relationships that could be construed as a potential conflict of interest.
